# Prescribing as a Lost Art

**Published:** 1909-09

**Authors:** 


Prescribing as a lost art.—That the pharmacopeia is an anachronism is asserted by an editorial writer in *The Hospital* (London, June 26). This, he says, is the reason why physicians prefer so often to use proprietary drugs rather than to write their own prescriptions, as of yore. Prescription writing is becoming a lost art because the "official" drugs are crude and variable in strength, while the active principles of those drugs can now be obtained pure, standardized, vouched for in both these respects by chemical firms of high scientific reputation, and convenient and easy to administer. Says the writer:

"Can it be wondered at that the medical practitioner of the present day finds himself driven, even against his material interest, to give the proved and pleasant forms of drug which the patient knows well enough are on the market, rather than to spend his time in learning the finesse of prescribing antiquated remedies? * * *

"The truth is that our pharmacopeia is an anachronism. It contains, of course, plenty of old and well-tried friends, but they are almost swamped in rubbish. When a person has traveled by an express train it is idle to assure him that a coach is the best way of getting about, and still more idle to complain, when he refuses to go back to coaching, that his tiresome choice is due to the fact that coachmen have forgotten how to drive. * * * No one is responsible for this except those whose business it is to keep the pharmacopeia level with the march of civilization. This they have neglected to do, and the result is what we see."—*Literary Digest.*

